# Supplementary material for: Metabolomic Analysis Reveals the Effect of Insecticide Chlorpyrifos on Rice Plant Metabolism
Source: Metabolites. 2022 Dec 19;12(12):1289. doi: 10.3390/metabo12121289 (PMC9786318; doi:10.3390/metabo12121289)
Supplement: Supplementary file 1 [file metabolites-12-01289-s001.zip › metabolites-2061717-supplementary.pdf]

Support information

Table S1 Information of MS peaks identified.

Time (min)	Average Mz	Mz Types	Metabolite	Class	Intensity			
					CK	Low	Middle	High
0.668	146.1652	[M+H]+	Spermidine[a]	Alkaloids	1.11E+03	3.87E+02*	1.23E+03	9.17E+02
0.729	191.0194	[M-H]-	Citric acid[a]	Organic acids	1.16E+06	1.30E+06*	1.05E+06*	1.19E+06
0.740	166.9731	[M-H]-	Phosphoenolpyruvic acid	Organic acids	6.59E+04	6.69E+04	8.70E+04*	9.02E+04*
0.744	131.1282	[M+H]+	Agmatine	Others	1.13E+03	1.29E+03	1.28E+03	9.34E+02*
0.761	133.0151	[M-H]-	Malic acid[a]	Organic acids	5.98E+05	5.82E+05	7.14E+05*	6.50E+05*
0.761	96.9609	[M-H]-	Phosphoric acid	Others	1.70E+07	1.37E+07*	1.59E+07*	1.58E+07*
0.770	185.0098	[M-H]-	2-Phosphoglycerate[a]	Others	2.77E+04	3.10E+04*	3.48E+04*	3.22E+04
0.783	259.0214	[M-H]-	D-Glucose 6-phosphate[a]	Sugars	1.40E+05	1.64E+05*	1.59E+05*	1.48E+05*
0.787	117.0189	[M-H]-	Succinic acid[a]	Organic acids	1.20E+04	1.15E+04	9.11E+03*	1.09E+04
0.792	147.0299	[M-H]-	Citramalic acid	Organic acids	4.16E+03	4.62E+03*	3.26E+03*	4.79E+03*
0.796	132.0308	[M-H]-	Aspartate[a]	Amino acids	3.14E+04	3.12E+04	3.41E+04*	2.74E+04*
0.797	188.0554	[M-H]-	N-acetyl-DL-glutamic acid	Amino acids	2.07E+03	1.59E+03*	1.39E+03*	1.70E+03*
0.799	191.0553	[M-H]-	Quinic acid[a]	Organic acids	5.63E+05	7.87E+05*	9.95E+05*	8.95E+05*
0.810	565.0465	[M-H]-	Uridine 5'-diphospho-D-glucose[a]	Nucleosides	1.13E+04	1.21E+04	1.16E+04	1.24E+04*
0.831	175.1173	[M+H]+	Arginine[a]	Amino acids	8.98E+02	1.58E+03*	1.40E+03*	1.35E+03*
0.860	131.0460	[M-H]-	Methylsuccinic acid[a]	Organic acids	4.57E+03	3.09E+03*	1.58E+03*	2.94E+03*
0.861	104.1058	[M]+	Choline[a]	Nucleosides	7.91E+04	7.82E+04	7.05E+04*	7.21E+04

0.861	104.0722	[M+H]+	N,N-dimethylglycine	Amino acids	7.69E+04	7.09E+04	6.74E+04*	6.73E+04*
0.886	130.0475	[M+H]+	L-5-oxoproline[a]	Amino acids	3.66E+04	3.79E+04	3.42E+04	2.54E+04*
0.893	148.0586	[M+H]+	Glutamic acid[a]	Amino acids	3.38E+03	3.46E+03	3.29E+03	2.82E+03*
0.901	156.0739	[M+H]+	Histidine[a]	Amino acids	1.40E+03	1.27E+03	1.82E+03*	1.60E+03
0.964	138.0539	[M+H]+	Trigonelline[a]	Alkaloids	3.53E+04	2.82E+04*	3.16E+04	2.90E+04*
0.976	116.0700	[M+H]+	Proline[a]	Amino acids	1.87E+04	1.75E+04	2.25E+04*	2.40E+04*
0.990	203.1490	[M+H]+	N,N-dimethylarginine	Amino acids	1.93E+03	1.42E+03	1.16E+03*	1.86E+03
0.994	527.1555	[M+NH4]+	Melezitose[a]	Sugars	3.10E+04	3.59E+04*	3.52E+04*	3.02E+04
0.995	365.1016	[M+Na]+	Melibiose[a]	Sugars	9.67E+03	1.35E+04*	1.19E+04*	1.07E+04
0.995	162.0763	[M+H]+	N-methyl-l-glutamic acid	Amino acids	7.74E+02	7.45E+02	5.21E+02	4.94E+02*
1.054	118.0868	[M+H]+	Betaine[a]	Alkaloids	1.87E+04	1.74E+04*	2.07E+04*	1.91E+04
1.188	152.0563	[M+H]+	Guanine[a]	Nucleosides	4.80E+02	7.75E+02*	5.50E+02	4.81E+02
1.415	123.0550	[M+H]+	Niacinamide[a]	Alkaloids	2.72E+04	2.85E+04*	2.55E+04	2.03E+04*
1.539	113.0339	[M+H]+	Uracil[a]	Nucleosides	3.95E+02	1.52E+03*	6.72E+02	3.10E+02
1.589	348.0685	[M+H]+	AMP[a]	Nucleosides	5.95E+04	5.73E+04	6.78E+04*	6.29E+04
1.683	308.0892	[M+H]+	Glutathione [a]	Amino acids	8.95E+03	1.48E+04*	1.13E+04*	1.30E+04*
1.755	123.0551	[M+H]+	Nicotinamide[a]	Alkaloids	2.74E+04	2.73E+04	2.40E+04	1.93E+04*
1.862	180.0679	[M-H]-	Tyrosine[a]	Amino acids	4.01E+04	4.05E+04	4.46E+04*	4.33E+04
2.060	104.0455	[M+H]+	Biuret	Others	5.22E+02	2.55E+02*	5.78E+02	3.52E+02*
2.069	664.1120	[M+H]+	NAD+[a]	Nucleosides	2.78E+03	5.53E+03*	2.27E+03	2.76E+03
2.102	132.1000	[M+H]+	Isoleucine[a]	Amino acids	9.84E+03	8.85E+03	8.59E+03*	8.69E+03

2.746	250.0714	[M-H]-	Phenylacetylaspartic acid	Amino acids	8.36E+02	1.59E+03	1.15E+03*	6.87E+02*
6.138	182.0434	[M-H]-	4-Pyridoxic acid	Vitamins	3.40E+04	3.27E+04	3.04E+04	2.66E+04*
6.461	220.1174	[M+H]+	Pantothenic acid[a]	Vitamins	6.70E+02	6.63E+02	8.50E+02*	7.27E+02
6.646	312.1256	[M+H]+	N2,N2-dimethylguanosine	Nucleosides	1.63E+03	1.72E+03	1.88E+03	1.22E+03*
6.994	367.1013	[M-H]-	3-Feruloylquinic Acid	Phenolic acids	4.18E+05	4.89E+05*	3.69E+05	3.38E+05*
7.112	205.0948	[M-H]-	Tryptophan[a]	Amino acids	3.61E+04	2.98E+04	3.23E+04	3.01E+04*
7.649	138.0548	[M+H]+	3-Aminobenzoic acid	Organic acids	3.32E+04	5.99E+04*	4.19E+04*	7.28E+04*
7.689	339.1056	[M+2K-H]+	3-O-coumaroylquinic Acid	Phenolic acids	7.96E+03	9.90E+03	1.03E+04*	9.73E+03
7.729	153.0199	[M-H]-	2,5-Dihydroxybenzoic acid	Organic acids	4.79E+05	4.77E+05	4.62E+05	4.48E+05*
7.743	291.0884	[M+H]+	Epicatechin[a]	Flavonoids	1.97E+04	2.38E+04*	1.68E+04	1.99E+04
8.268	307.1758	[M+H]+	Feruloyl agmatine	Phenolic acids	1.24E+03	3.22E+03*	3.99E+02*	5.53E+02*
8.448	377.1448	[M+H]+	Riboflavin[a]	Vitamins	1.59E+03	2.21E+03*	1.33E+03	1.27E+03
8.624	121.0302	[M-H]-	4-Hydroxybenzaldehyde	Others	6.79E+04	7.01E+04	8.13E+04*	6.85E+04
8.666	595.1659	[M+H]+	Biotin	Flavonoids	7.64E+04	7.46E+04	5.60E+04*	6.22E+04*
8.704	609.1668	[M-H]-	Hesperetin 7-O-Rutinoside	Flavonoids	1.55E+06	1.35E+06*	1.21E+06*	1.23E+06
8.745	563.1425	[M-H]-	Corymboside	Flavonoids	7.11E+06	6.83E+06	6.45E+06	6.35E+06*
8.845	449.1079	[M+H]+	homoorientin[a]	Flavonoids	7.43E+05	7.37E+05	7.10E+05	6.02E+05*
8.902	449.1036	[M+H]+	Isoorientin[a]	Flavonoids	6.82E+05	7.30E+05	6.88E+05	6.08E+05*
8.907	565.1561	[M+H]+	Schaftoside	Flavonoids	2.29E+06	2.55E+06	2.23E+06	2.12E+06*
8.947	207.0116	[M-H]-	2-Naphthalenesulfonic acid	Organic acids	1.84E+02	2.07E+02	1.52E+02	1.48E+02*
9.085	447.0956	[M-H]-	Luteolin 6-C-glucoside	Flavonoids	7.99E+05	5.77E+05	7.17E+05*	6.15E+05*

9.157	433.1048	[M+H]+	Isovitexin	Flavonoids	2.32E+04	2.78E+04*	2.30E+04	2.68E+04
9.286	625.1759	[M+H]+	Iscopalin 2-O-glucoside	Flavonoids	6.16E+06	6.49E+06	5.30E+06*	5.54E+06*
9.320	579.1732	[M+H]+	Vitexin 2-O-rhamnoside	Flavonoids	1.29E+04	1.70E+04*	1.20E+04	1.55E+04
9.734	479.1227	[M+H]+	Isorhamnetin 3-O-glucoside	Flavonoids	3.01E+05	3.23E+05	2.53E+05	2.27E+05*
9.874	491.1159	[M-H]-	Tricin 5-glucoside	Flavonoids	4.15E+06	3.76E+06	3.59E+06	3.56E+06*
9.925	463.1179	[M+H]+	Hispidulin 4-glucoside	Flavonoids	6.14E+05	5.83E+05	8.23E+05*	4.78E+05*
10.017	579.1663	[M+H]+	Apigenin 7-O-neohesperidoside	Flavonoids	9.77E+04	8.05E+04	6.04E+04*	7.11E+04*
10.155	721.2123	[M+FA-H]-	Icariin	Flavonoids	1.05E+05	8.30E+04*	6.61E+04*	9.28E+04
10.290	431.0978	[M-H]-	Genistin[a]	Flavonoids	3.73E+04	3.31E+04	2.73E+04*	2.97E+04*
10.307	639.1921	[M+H]+	Demethoxycentaureidin 7-O-rutinoside	Flavonoids	6.28E+06	7.28E+06*	5.80E+06*	6.47E+06
10.497	144.0460	[M-H]-	Indole-3-carboxaldehyde[a]	Others	1.02E+04	1.19E+04	1.07E+04	9.07E+03*
10.549	757.2097	[M]+	Cyanidin 3-(2G-glucosylrutinoside)	Flavonoids	7.59E+04	7.10E+04	6.64E+04*	5.62E+04*
10.636	463.1209	[M]+	Peonidin 3-O-glucoside	Flavonoids	4.90E+05	4.13E+05*	3.60E+05*	3.83E+05*
10.726	515.1521	[M+H]+	Globularin	Others	2.92E+05	2.85E+05	2.63E+05*	3.00E+05
11.536	314.1367	[M+H]+	feruloyltyramine	Phenolic acids	1.74E+04	1.20E+04*	1.30E+04*	1.44E+04*
11.758	257.0794	[M+H]+	Glycyrrhizin	Flavonoids	4.58E+03	4.89E+03	5.08E+03	5.76E+03*
13.055	331.0817	[M+H]+	Tricin	Flavonoids	1.01E+06	1.26E+06*	8.49E+05*	1.27E+06*
16.778	483.2626	[M-H]-	LPG 16:0	Lipids	1.41E+05	1.24E+05	1.77E+05*	1.20E+05
17.160	308.2218	[M+H]+	Dihydrocapsaicin	Others	3.40E+03	3.50E+03	3.57E+03	3.87E+03*
17.180	325.1845	[M-H]-	Hydroquinidine	Others	1.81E+05	2.09E+05	1.92E+05	2.32E+05*
17.658	478.2907	[M+H]+	LPE 18:2	Lipids	1.58E+05	1.58E+05	1.99E+05*	1.50E+05

17.991	415.2055	[M-H2O+H]+	Gelomulide N	Others	3.94E+02	7.18E+02*	7.59E+02*	7.17E+02
18.568	454.2911	[M+H]+	LPE 16:0	Lipids	9.60E+04	1.12E+05*	1.09E+05*	9.69E+04
19.100	480.3062	[M-H]-	LPE 18:1	Lipids	7.33E+03	8.97E+03*	1.14E+04*	7.90E+03*
19.213	522.3542	[M+H]+	LPC 18:1	Lipids	9.81E+04	1.20E+05*	1.47E+05*	1.03E+05
19.264	247.1635	[M-H]-	FA 16:4	Lipids	3.08E+04	3.45E+04*	3.50E+04*	3.24E+04
19.530	468.3101	[M+H]+	LPE 17:0	Lipids	2.67E+03	3.48E+03	3.32E+03*	3.49E+03*
20.067	510.3586	[M+H]+	LPC 17:0	Lipids	3.52E+04	3.59E+04	4.63E+04*	3.93E+04
21.569	277.2196	[M+H]+	FA 18:3 (Linolenic acid) [a]	Lipids	8.76E+03	1.25E+04*	1.20E+04*	1.15E+04*
21.640	267.2205	[M-H]-	FA 17:1	Lipids	3.03E+03	2.66E+03	2.98E+03	4.03E+03*
22.039	728.5250	[M-H]-	PE (O-18:0/18:2)	Lipids	4.12E+03	3.62E+03	2.43E+03*	3.51E+03
22.273	301.2113	[M-H]-	FA 20:5	Lipids	3.47E+03	1.95E+03*	1.19E+04*	1.56E+04*
22.680	855.4913	[M-H]-	PI (18:2/18:3)	Lipids	1.26E+05	1.42E+05*	1.59E+05*	1.50E+05
22.754	765.4463	[M-H]-	PG (18:3/18:3)	Lipids	1.21E+05	1.16E+05	1.23E+05	1.38E+05*
22.941	317.2402	[M-H]-	FA 21:4	Lipids	4.18E+02	3.22E+02	4.27E+02	3.30E+02*
23.041	738.4948	[M-H]-	PE (18:2/18:2)	Lipids	5.00E+05	4.45E+05*	5.58E+05*	5.92E+05*
23.145	281.2461	[M+H]+	FA 18:2 (Linoleic acid) [a]	Lipids	1.86E+06	2.63E+06*	2.86E+06*	3.16E+06*
23.198	831.4882	[M-H]-	PI (16:0/18:3)	Lipids	9.52E+04	1.13E+05*	1.23E+05*	1.21E+05*
23.241	857.4995	[M-H]-	PI (18:2/18:2)	Lipids	2.42E+05	2.94E+05*	4.35E+05*	4.42E+05*
23.399	729.4594	[M-H]-	PG (15:0/18:3)	Lipids	3.82E+05	4.26E+05	4.32E+05*	4.41E+05*
23.469	307.2573	[M-H]-	FA 20:2	Lipids	3.21E+03	3.38E+03	4.17E+03*	5.94E+03

23.516	705.4363	[M-H]-	PMeOH (18:3/18:3)	Lipids	3.39E+05	4.71E+05*	2.60E+05*	2.91E+05
23.554	269.2509	[M-H]-	FA 17:0	Lipids	2.51E+04	2.57E+04	2.78E+04*	2.52E+04
23.775	833.5386	[M-H]-	PI (16:0/18:2)	Lipids	6.34E+06	7.24E+06	1.06E+07*	1.06E+07*
23.795	655.4262	[M-H]-	PMeOH (14:0/18:3)	Lipids	2.70E+04	3.75E+04*	1.98E+04*	2.14E+04*
23.827	743.4689	[M-H]-	PG (16:0/18:3)	Lipids	4.21E+07	4.57E+07*	4.65E+07*	4.88E+07*
23.859	769.4881	[M-H]-	PG (18:2/18:2)	Lipids	1.09E+06	1.18E+06*	1.29E+06*	1.23E+06*
23.922	859.5203	[M-H]-	PI (18:1/18:2)	Lipids	9.83E+04	1.15E+05*	2.05E+05*	2.04E+05*
23.985	731.5120	[M-H]-	PG (15:0/18:2)	Lipids	7.98E+05	8.30E+05	8.84E+05*	9.62E+05*
23.985	753.4936	[M-H]-	PG (O-18:3/18:2)	Lipids	2.89E+04	3.44E+04*	3.96E+04*	4.39E+04*
24.133	672.5064	[M-H]-	PE (O-14:0/18:2)	Lipids	1.81E+04	1.77E+04	1.25E+04*	1.53E+04*
24.155	707.4547	[M-H]-	PMeOH (18:2/18:3)	Lipids	1.82E+06	2.44E+06*	1.38E+06*	1.42E+06*
24.313	719.4650	[M-H]-	PG (16:0/16:1)	Lipids	3.14E+07	3.35E+07	3.68E+07	3.92E+07*
24.345	757.5089	[M-H]-	PG (17:0/18:3)	Lipids	2.80E+05	3.06E+05	4.29E+05*	4.82E+05*
24.345	669.4441	[M-H]-	PMeOH (15:0/18:3)	Lipids	1.20E+05	1.70E+05*	1.15E+05	1.24E+05
24.520	657.4400	[M-H]-	PMeOH (14:0/18:2)	Lipids	2.04E+04	2.99E+04*	2.20E+04	2.17E+04
24.524	847.5261	[M-H]-	PI (17:0/18:2)	Lipids	7.80E+04	7.31E+04	1.50E+05*	1.84E+05*
24.598	745.4914	[M-H]-	PG (16:0/18:2)	Lipids	2.23E+07	2.75E+07*	2.93E+07*	3.10E+07*
24.725	771.5064	[M-H]-	PG (18:1/18:2)	Lipids	1.60E+04	1.97E+04	3.06E+04*	3.28E+04*
25.005	309.2740	[M-H]-	FA 20:1	Lipids	5.60E+04	6.04E+04*	6.76E+04*	7.64E+04

[a] Confirmed by the standard database; * metabolites with significant differences compared to the control.
